# A Retrospective Review of Native Septic Arthritis in Patients: Can We Diagnose Based on Laboratory Values?

**DOI:** 10.7759/cureus.8577

**Published:** 2020-06-12

**Authors:** Luke Rasmussen, Jared Bell, Arun Kumar, Michael G Heckman, Elizabeth Lesser, Joseph Whalen, Glenn G Shi, Cameron Ledford, Benjamin Wilke

**Affiliations:** 1 Orthopedics, Mayo Clinic, Jacksonville, USA; 2 Biostatistics, Mayo Clinic, Jacksonville, USA; 3 Emergency Medicine, Mayo Clinic, Jacksonville, USA

**Keywords:** septic arthrits, cell count

## Abstract

Introduction

The accurate diagnosis of acute septic arthritis is essential to initiating appropriate treatment and minimizing potential cartilage damage. A synovial fluid cell count of 50,000 cells/mm^3^ has been used as a diagnostic cutoff for acute septic arthritis, although data supporting this is lacking. The purpose of this study was to assess the efficacy of synovial cell counts to predict septic arthritis in patients with symptomatic native joints.

Methods

A retrospective review was performed of patients who were evaluated for septic arthritis at a single institution with the use of synovial fluid analysis and adjunctive lab tests. Exclusion criteria included history of a total joint arthroplasty of the affected joint or immunocompromised state. A true infection was considered on the basis of positive or negative synovial aspirate cultures. We evaluated the synovial cell count, synovial polymorphonuclear cell percentile (% neutrophils), serum white blood cell (WBC), erythrocyte sedimentation rate (ESR), and C-reactive protein (CRP) in order to determine their association and predictive power in a true infection.

Results

Of the 65 patients included in the study, 40 (61.5%) had a positive culture for septic arthritis and 25 (38.5%) had negative cultures. Patients with positive cultures had a larger median % neutrophils than patients with negative cultures (median: 93 vs. median: 86, P=0.041). They also tended to have higher serum CRP levels compared to negative culture patients (median: 142.30 vs. 34.20, P=0.051). No outcomes were independently highly effective in discriminating between patient groups (area under the curve (AUC) ≤ 0.67). There was no significant difference between the synovial cell counts in patients with culture positive septic arthritis and patients with negative cultures (median: 32435 vs 35385, P = 0.94).

Conclusion

Patients with culture proven septic arthritis had larger % neutrophils. However, there were no other statistically significant differences between patient groups regarding ESR, CRP, WBC, or cell count aspiration at the time of diagnosis. No synovial cell count level was highly effective in discriminating patients with a positive culture for septic arthritis from patients with negative cultures.

## Introduction

Native septic arthritis remains a common and morbid disease. In the United States, the estimated annual incidence of septic arthritis varies from 2-10 per 100,000 people, and of adults who present to Emergency Departments with acute joint pain, approximately 27% are diagnosed with septic arthritis [1,2]. If not treated promptly, septic arthritis can lead to significant joint destruction, acute kidney injury, sepsis, chronic infection, and a mortality up to 11% [3].

To avoid complications, septic arthritis must be rapidly and accurately diagnosed with early initiation of treatment, which often involves surgical debridement. Further, pathologies such as crystalline and inflammatory arthropathies may present with similar clinical and laboratory findings. These pathologies do not typically require surgical intervention, so the clinician must be able to differentiate between these in order to prevent the under-treatment of septic arthritis or over-treatment of crystalline or inflammatory arthritis.

The diagnosis of septic arthritis has historically been made using clinical examination and laboratory data. The gold standard test is a positive tissue or fluid culture taken directly from the affected joint; however, this result may take several days, delaying care to the patient and increasing the potential destruction to the joint. Clinicians, therefore, must rely on more rapidly obtained lab testing, such as the synovial fluid white cell count to determine if a patient has septic arthritis. It is traditionally accepted that a synovial fluid cell count of 50,000 cells/mm3 or higher, isolated from a native joint, is diagnostic of septic arthritis with lower counts suggestive of a crystalline or inflammatory arthropathy [4-8]. While this cutoff is often utilized, the evidence to support such a value is relatively lacking. Previous studies that have reported this value have used various definitions for septic arthritis, clouding the diagnostic picture. The primary objective of this research was to review patients who have had a positive culture from a joint aspiration and compare their synovial fluid cell count values to determine if the 50,000 cells/mm3 cutoff is truly reliable.

## Materials and methods

After Institutional Review Board approval, this retrospective cohort study was performed at a single institution and included all patients diagnosed with septic arthritis between February 2008 and August 2018. A search identified 490 patients by International Classification of Diseases, 9th Revision (ICD-9) Icodes 711.0 and 711.9 (pyogenic arthritis, and unspecified infective arthritis, respectively) and ICD 10 code M00.9 (pyogenic arthritis, unspecified). The patients were then screened to exclude those who did not undergo an aspiration, those with septic arthritis involving a joint replacement, those that took antibiotics prior to the aspiration and had a subsequent negative culture, and those patients with a diagnosis of an immunodeficiency (defined as transplant patients on anti-rejection medication, cancer patients receiving chemotherapy, or patients with an inflammatory disorder receiving immune-modulating medications). Patients were included if they had confirmed culture-positive aspiration confirming septic arthritis in a native joint. In total, 40 patients met the criteria and were then contrasted to a comparison group of 25 patients with a culture-negative aspiration, who also had not received antibiotics prior to the aspiration.

Data from the time of the patient’s initial evaluation was collected, including patient age, sex, affected joint, and laterality. Select diagnostic laboratory values including the erythrocyte sedimentation rate (ESR), C-reactive protein (CRP), and white blood cell count (WBC) were reviewed. Additional data included synovial fluid cell count, percent of synovial fluid polymorphonuclear cells (% neutrophils), culture results, and whether the patient subsequently underwent a debridement operation.

Continuous variables were summarized with median and range, and categorical variables were summarized with frequency and percent. Continuous variables were compared between patients with a positive and negative culture for septic arthritis using a Wilcoxon Rank Sum test, whereas categorical variables were compared between these two groups using a Pearson chi-square test. Additionally, to evaluate the ability of ESR at diagnosis, CRP at diagnosis, WBC at diagnosis, and cell count and % neutrophils from the aspiration to discriminate between patients with a positive culture for septic arthritis and those with a negative culture, we estimated area under the receiver operating characteristic (ROC) area under the curve (AUC) along with 95% confidence intervals (CIs). An AUC of 1.0 indicates perfect predictive ability whereas and AUC of 0.5 indicates predictive ability equal to chance. All statistical tests were two-sided and p-values less than 0.05 were considered statistically significant. All statistical analyses were performed in R Statistical Software, version 3.4.2 (R Foundation for Statistical Computing, Vienna, Austria).

## Results

There were 40 males (61.5%) and 25 females (38.5%) in the total cohort. The average age was 66 years (19-96 years). Knees were the most commonly affected joints followed by hips, shoulders, ankles, elbows, and wrists. Patient characteristics are summarized in Table 1. 

**Table 1:** Patient characteristics according to culture for septic arthritis (negative or positive)

	Negative (N=25)	Positive (N=40)	p-value
Age at diagnosis	67 (19, 95)	66 (21, 96)	0.19
Males	11 (44.0%)	29 (72.5%)	0.022
Affected Joint (knee / hip / shoulder)			0.95
Ankle	3 (12.0%)	5 (12.5%)	
Elbow	0 (0.0%)	1 (2.5%)	
Hip	6 (24.0%)	7 (17.5%)	
Knee	12 (48.0%)	21 (52.5%)	
Shoulder	3 (12.0%)	5 (12.5%)	
Wrist	1 (4.0%)	1 (2.5%)	
Right laterality	14 (56.0%)	21 (52.5%)	0.78
Treated operatively	13 (52.0%)	39 (97.5%)	<0.001
Received antibiotics prior to aspiration	0 (0.0%)	12 (30.0%)	0.002
Continuous variables are summarized with median (range), and categorical variables are summarized with number (%).P-values less than 0.05 are considered statistically significant and are shown in bold.			

Of the 65 patients, 40 (61.5%) had a culture-positive aspirate, and 25 (38.5%) had a negative aspirate culture. Patients who had a positive culture for septic arthritis were a higher proportion of males than patients with a negative culture (72.5% vs. 44.0%, P=0.022). As expected, antibiotic use prior to aspiration and operative treatment differed between the two groups (Table 1). No other patient characteristic differences were observed between the cohorts.

Thirty-nine patients in the culture positive cohort (97.5%) underwent an irrigation and debridement procedure. One medically complex patient was treated without formal surgical debridement due to high risk of mortality associated with anesthesia and the surgical procedure.

**Figure 1:**
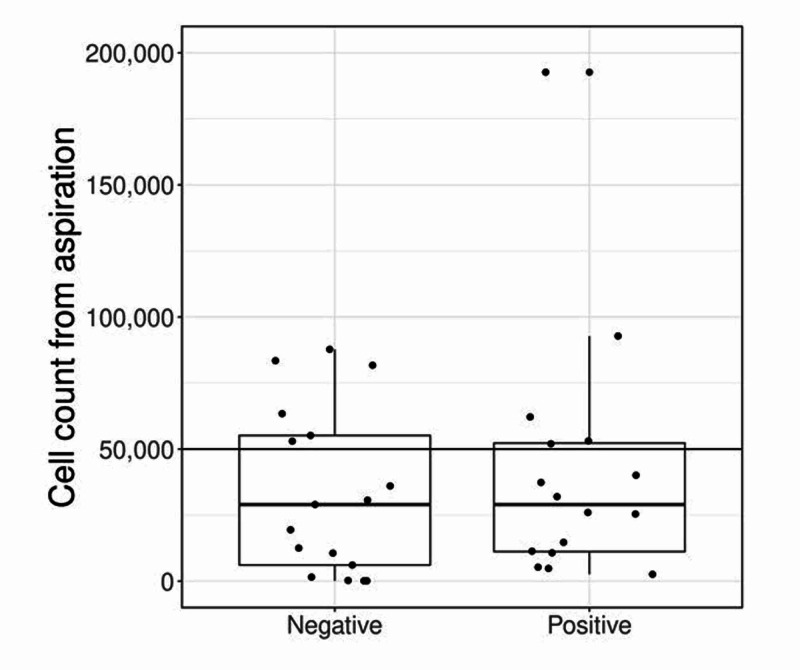
Synovial fluid cell count between groups

Laboratory values are compared between patient groups in Table 2 and Figure 1. Culture results are presented in Table 3. Culture-positive patients demonstrated a larger percentage of synovial polymorphonuclear cells (% neutrophils) than patients with a negative culture (median: 93% vs. median: 86%, P=0.041). These patients also had increased ESR, CRP, and WBC values compared to the culture-negative cohort; however, these differences were not statistically significant with the exception of CRP, which did approach significance. Additionally, no difference in synovial fluid cell count between the culture-positive and culture-negative patients (median: 35,385 and 32,435 respectively p=0.94). Out of 40 patients with culture-positive septic arthritis, only 13 (32.5%) had a synovial cell count > 50,000 cells/mm3. No studied outcomes were highly effective in discriminating between patient groups (AUC ≤ 0.67).

**Table 2:** Laboratory values according to culture for septic arthritis status

	Negative (N=25)	Positive (N=40)	AUC (95% CI)	p value
% Neutrophils	86 (4, 98)	93 (5, 100)	0.65 (0.52, 0.79)	0.041
ESR at Diagnosis	32 (1, 120)	49.5 (2, 145)	0.61 (0.44, 0.78)	0.21
CRP at diagnosis	34.20 (1.10, 352.60)	142.30 (2.60, 400.0)	0.66 (0.50, 0.83)	0.051
WBC at diagnosis	8.89 (4.00, 20.30)	10.00 (5.20, 15.40)	0.51 (0.34, 0.68)	0.89
Cell count from aspiration	35385 (157, 333000)	32435 (33, 260000)	0.51 (0.36, 0.66)	0.94
CI=confidence interval, ESR = erythrocyte sedimentation rate, CRP = C-reactive protein, WBC = white blood cell count. Continuous variables are summarized with median (range), and categorical variables are summarized with number (%). Twelve patients were missing ESR at diagnosis, fifteen patients were missing CRP at diagnosis, and four patients were missing WBC at diagnosis. P-values less than 0.05 are considered statistically significant and are shown in bold.				

**Table 3:** Bacterial isolate percentiles in culture positive joints

	Isolates (N=43)		Isolate frequency (% of aspirates)
Methicillin resistant Staphylococcus aureus	15		(37.5%)
Staphylococcus aureus	13		30.0%
Group B Streptococci	3		7.5%
Coagulase-Negative Staphylococci	2		5.0%
Pseudomonas aeruginosa	1		2.5%
Streptococcus viridans	1		2.5%
Streptococcus pneumoniae	1		2.5%
Propionibacerium acnes	1		2.5%
Group G Streptococci	1		2.5%
Serratia marcescens	1		2.5%
Diphtheroid bacilli	1		2.5%
Ralstonia picketti	1		2.5%
Caulobacter Sp.	1		2.5%
Verticillium Sp.	1		2.5%
Mycobacterium avium	1		2.5%
43 isolates were identified from 40 aspirate samples. Isolate frequency is expressed as the percentage of aspirates that cultured the identified isolate (N =40).			

## Discussion

The diagnosis of septic arthritis continues to be a challenging task for clinicians. Delayed diagnosis may lead to irreversible joint damage while over-diagnosis may cause patients to undergo unnecessary medical and surgical treatments; therefore, accurate diagnosis is crucial [9]. The gold standard diagnostic test remains a positive culture from the affected joint. As cultures often take days to produce results, clinicians must rely on alternate laboratory data in order to initiate prompt treatment. In addition to common blood tests such as the ESR, CRP, and CBC, the synovial fluid cell count and percentage of synovial fluid polymorphonuclear cells from the joint aspiration is perhaps the most critical determinant of native septic arthritis. A synovial fluid cell count of 50,000 cells/mm3 or higher is typically concerning for septic arthritis in a native joint, while lower values are more consistent with a crystalline or inflammatory arthropathy [4-8,10]. However, the literature to support this cutoff is rather limited, yet this value is treated in a dogmatic manner. 

Previous reports have used varying definitions for septic arthritis; while synovial cultures are the gold standard, they are estimated to be only 75%-95% sensitive [5,6,11]. It is thought that if synovial cultures are used in isolation then some instances of septic arthritis may be missed [4,12]. In 1976, Newman defined septic arthritis as being present if one of the following criteria were met: 1) an organism isolated from the affected joint, 2) an organism isolated from elsewhere with a clinically swollen, painful joint, 3) no organism isolated, but histologic or radiologic evidence of infection, and 4) turbid fluid aspirated from the joint in a patient that has previously received antibiotics [11]. This definition remains popular today and is often used in lieu of positive cultures [5].

In the present study, those patients with culture-positive septic arthritis were included (rather than those that strictly fit Newman’s definition) to limit the inclusion of false positive patients. In this manner we likely missed cases of septic arthritis; the purpose, however, was not to determine the prevalence of septic arthritis at our institution, but rather to determine the synovial cell count in patients with true septic arthritis. Likewise, in the comparison group, we excluded all patients that had a negative culture who had received antibiotics prior to the aspiration due to the possibility that this may have masked the infection. While this does not serve as a perfect comparison group (as many of these patients still underwent treatment for presumed septic arthritis), their data is useful to contrast against patients with culture-positive septic arthritis.

Previous studies have attempted to evaluate the diagnostic power of laboratory findings in septic arthritis and have reported conflicting results [5,6,13-19]. For example, a systematic review by Margaretten et al. evaluated 14 studies in order to determine the most accurate laboratory evaluation for diagnosing septic arthritis [5]. Their criteria for diagnosing septic arthritis was similar to Newman’s, including patients with positive cultures as well as those that responded to antibiotics. They reported that the synovial cell count and percentage of polymorphonuclear cells in the aspiration were the most powerful tests for septic arthritis. A synovial cell count of 25,000 - 50,000 cells/mm3 had a likelihood ratio of 2.9 for septic arthritis, whereas a cell count of >50,000 cells/mm3 had a likelihood ratio of 7.7. Similarly, patients with >90% neutrophils in the aspiration had a likelihood ratio of 3.4, compared to 0.34 for those with <90% neutrophils in the aspirate. They found that joint pain was 85% sensitive and swelling was 78% sensitive, but recommended relying on the lab tests more than physical examination [5]. A separate study using similar diagnostic criteria reported a lower likelihood ratio of 1.06 if the cell count was 25,000 - 50,000, rising to 3.59 once the cell count was above 50,000 cells/mm3 [13].

In contrast to the two previous studies, Li et al. performed a retrospective review of 156 patients who underwent arthrocentesis. Their diagnostic criteria for septic arthritis included only those with positive cultures or intraoperative findings suggestive of infection. They reported that a cell count of 50,000 had a sensitivity of only 50% and instead recommended a cutoff of 17,500 in order to maximize the sensitivity (83%) and specificity (67%). They recommended using this cutoff to assist in ruling out septic arthritis rather than diagnosing it [6].

In our study, we found no statistically significant difference in the average cell count between the culture-positive and culture-negative cohorts. We also found that most patients with culture-positive septic arthritis had a synovial cell count that was below the 50,000 cells/mm3 cutoff that is been reported in the literature. Moreover, the synovial cell count was found to be ineffective in predicting septic arthritis (AUC 0.51, P=0.94).

Our study additionally demonstrated that the percentage of polymorphonuclear cells in the synovial fluid was one of the most accurate laboratory tests for predicting septic arthritis, similar to the study by Margaretten et al. [5]. The results of this study disagree with Margaretten’s conclusion that clinicians should rely on laboratory testing rather than physical examination to diagnose septic arthritis as none of the laboratory tests proved to have a high reliability as based on the AUC ≤ 0.67.

There were several limitations with this study. It was a retrospective review and has all the weaknesses inherent with a retrospective study design. Multiple joints were included which may have different physiologic responses to infection, however, previous studies have also included multiple joints in the study design [16-17]. The main weakness was the small sample size and was largely due to our strict criteria for diagnosing septic arthritis. Despite these limitations, we believe the data is valuable as this helps us better define the importance of the synovial cell count when attempting to diagnose septic arthritis.

## Conclusions

In conclusion, the vast majority of immunocompetent patients with culture-positive septic arthritis treated at our institution had a synovial cell count less than the historical recommended cutoff of 50,000 cells/mm3. We recommend relying less on the absolute synovial cell count and more heavily on the complete clinical picture, including history, physical examination, variety of laboratory values with emphasis on the percentage of polymorphonuclear cells in the synovial fluid in order to most promptly diagnose and treat patients with native septic joint arthritis. 
